# Supplementary material for: Predicting the structural impact of human alternative splicing
Source: Genome Biol. 2025 Sep 17;26:283. doi: 10.1186/s13059-025-03744-x (PMC12442299; doi:10.1186/s13059-025-03744-x)
Supplement: Supplementary file 1 — Additional file 1: Supplementary Fig. S1-S12. [file 13059_2025_3744_MOESM1_ESM.docx]

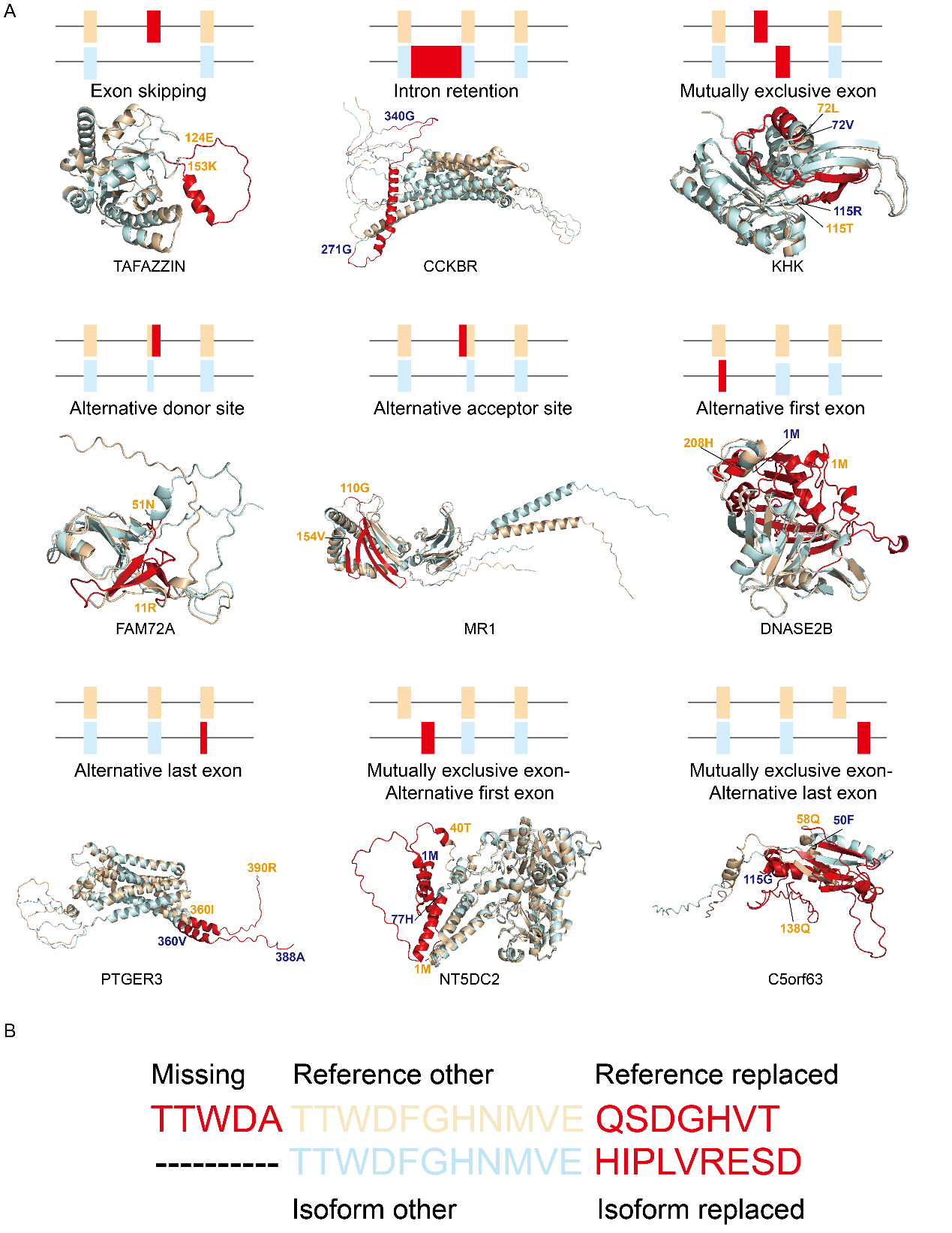


**Fig. S1: Illustration of nine alternative splicing types and different alternative splicing regions.** (**A**) Diagram for nine alternative splicing types represented by transcripts and example structures. For each type, reference and isoform structures are colored in wheat and pale cyan, respectively. Alternatively spliced regions are shown in red. The start and end alternative splicing regions are colored in orange (reference) and blue (isoform). (**B**) Explanation of spliced and unspliced regions. Splicing regions including ‘Missing’, ‘Reference replaced’ and ‘Isoform replaced’ are colored in red, and unspliced regions (‘Reference other’ and ‘Isoform other’) are presented in wheat and pale cyan, respectively.


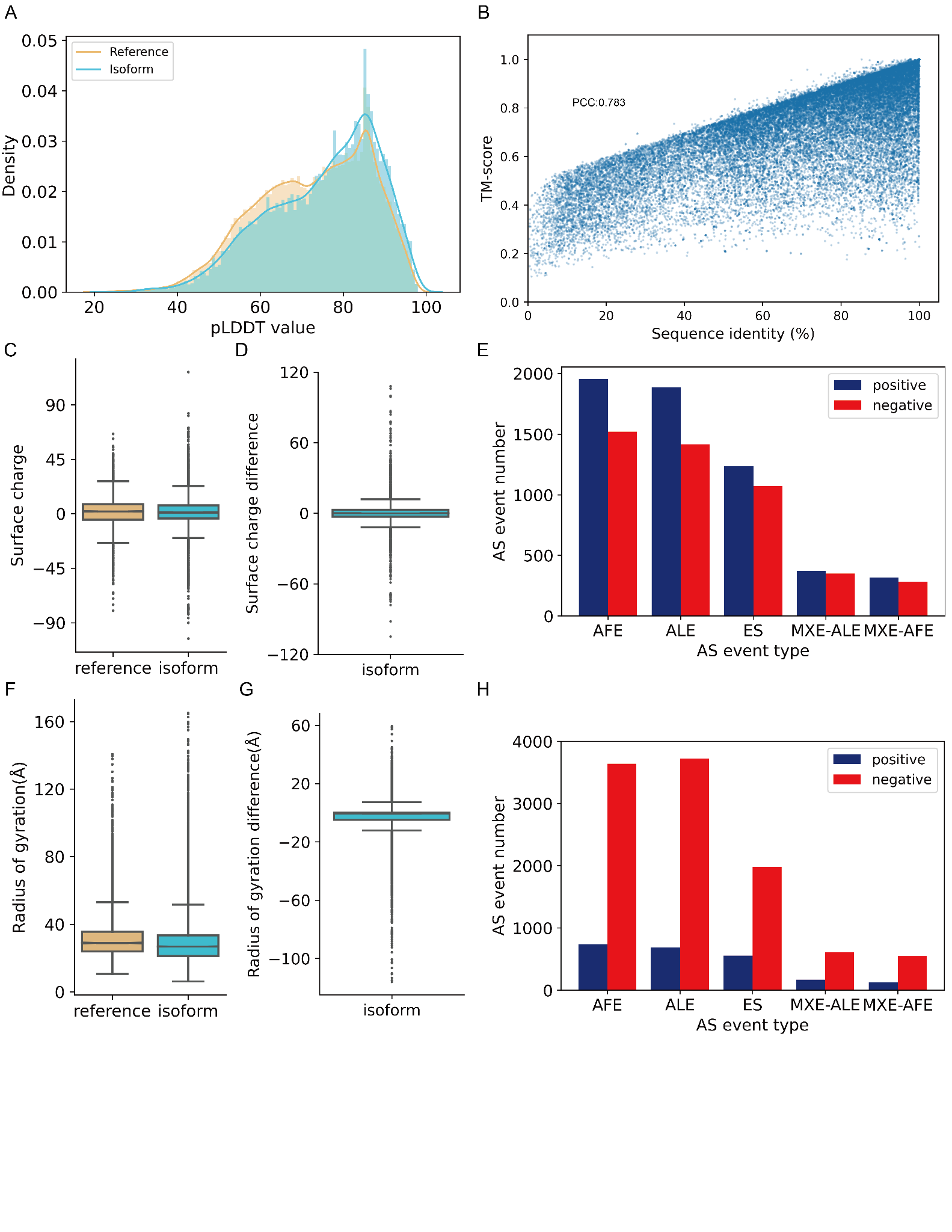


**Fig. S2: Structural metrics analysis for the CHESS dataset.** (**A**) Distribution of prediction quality for 15,727 reference and 9,7824 isoform structures from the CHESS dataset. (**B**) Scatter plot of percent sequence identity between reference and alternate isoform (x-axis) vs. TM score (y-axis) for the CHESS dataset. (PCC=0.783). (**C**) Overall surface charge distribution between reference and isoform structures from the CHESS dataset (p-value: 6.053e-11, Mann–Whitney U test). (**D**) Differences of surface charge for the CHESS dataset. (**E**) The five most frequent alternative splicing events in the positive and negative surface charge outliers from the CHESS dataset. (**F**) Overall radius of gyration distribution between reference and isoform structures from the CHESS dataset (p-value: 1.074e-115, Mann–Whitney U test). (**G**) Differences of radius of gyration from the CHESS dataset. (**H**) The five most frequent alternative splicing events in the positive and negative radius of gyration outliers from the CHESS dataset.


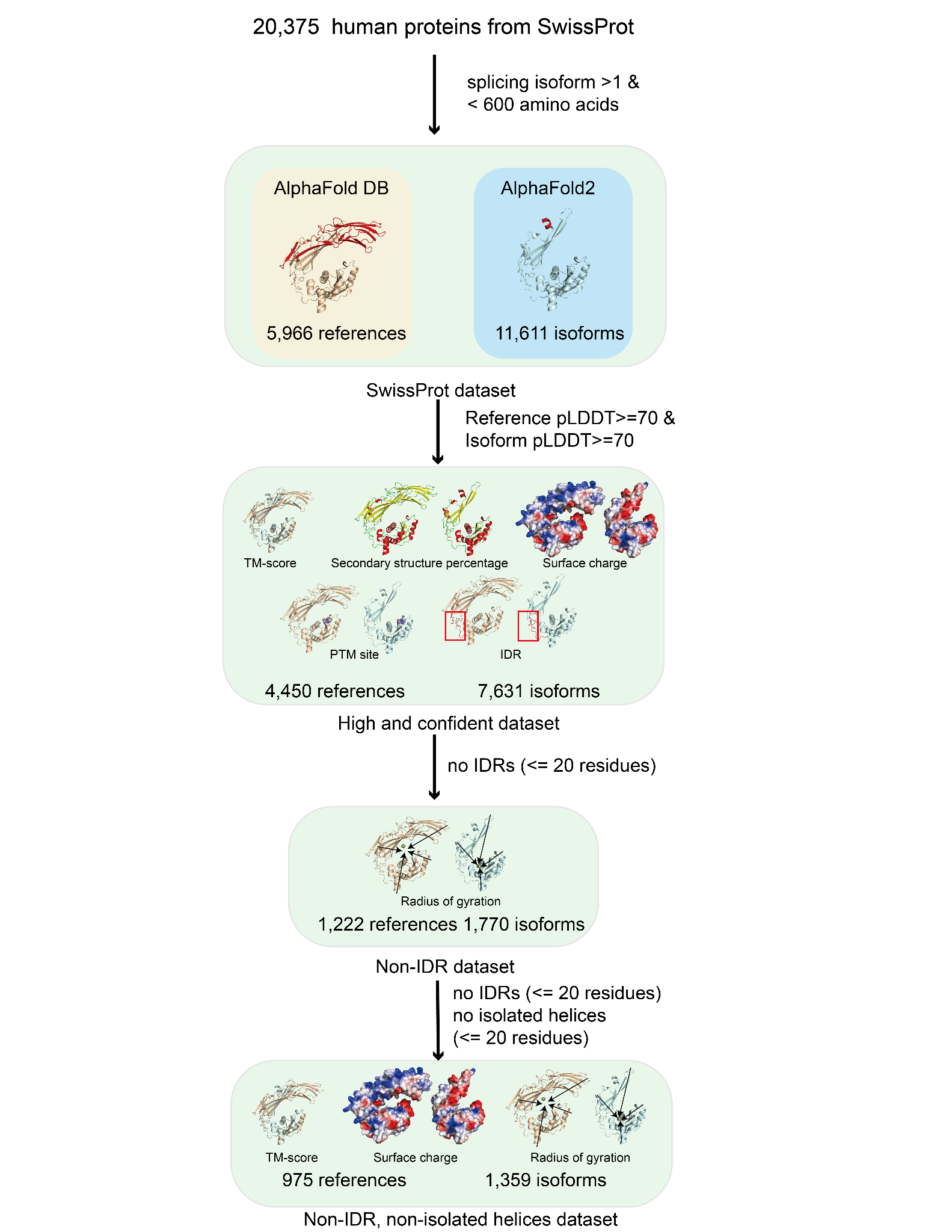


**Fig. S3: Flowchart of dataset filtering process.** Starting from the SwissProt human dataset, successive filtering steps yield four datasets used in this study: the SwissProt dataset, the high and confident dataset, the Non-IDR dataset and the non-IDR, non-isolated helices dataset. Each filtering step is annotated with its corresponding criteria, and the number of remaining structures along with their associated analyses is indicated.


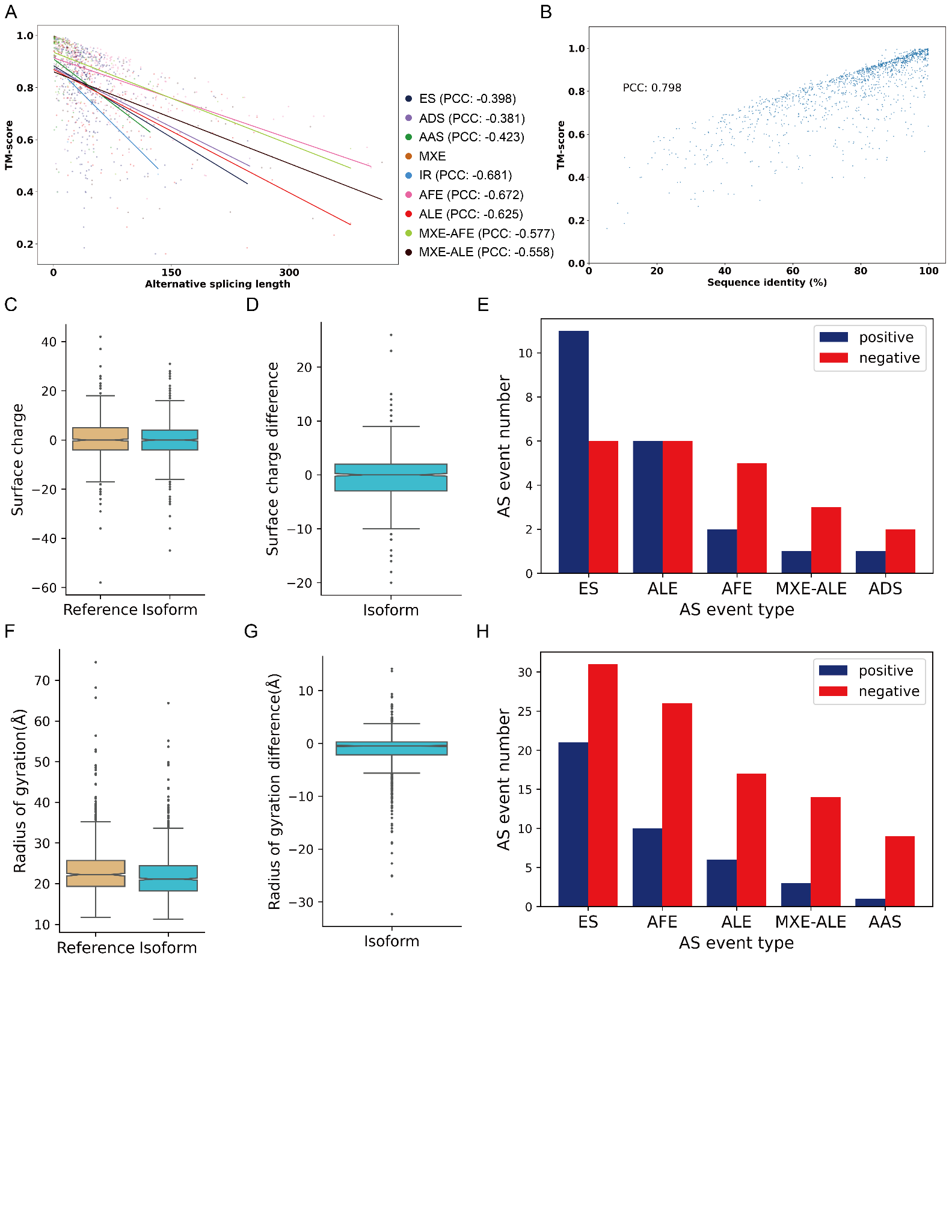


**Fig. S4: Structural metrics analysis for the non-IDR, non-isolated helices dataset.** (**A)** Scatter plot of length of alternative splicing region (x-axis) vs. TM-score for 999 alternate isoforms with their reference structures (y-axis) colored by alternative splicing type, fitted lines are presented for each alternative splicing type. (**B**) Scatter plot of percent sequence identity between reference and alternate isoform (x-axis) vs. TM score (y-axis) (PCC=0.798, N=1,359). (**C**) Overall surface charge distribution between reference and isoform structures (p-value: 0.58, Mann–Whitney U test, N=1,359). (**D**) Differences of surface charge from the non-IDR, non-isolated helices dataset, N=1,012. (**E**) The five most frequent alternative splicing events in the positive and negative surface charge outliers. (**F**) Overall radius of gyration distribution between reference and isoform structures (p-value: 9.497e-9, Mann–Whitney U test, N=1,359). (**G**) Differences of radius of gyration from the non-IDR, non-isolated helices dataset, N=1,012. (**H**) The five most frequent alternative splicing events in the positive and negative radius of gyration outliers.


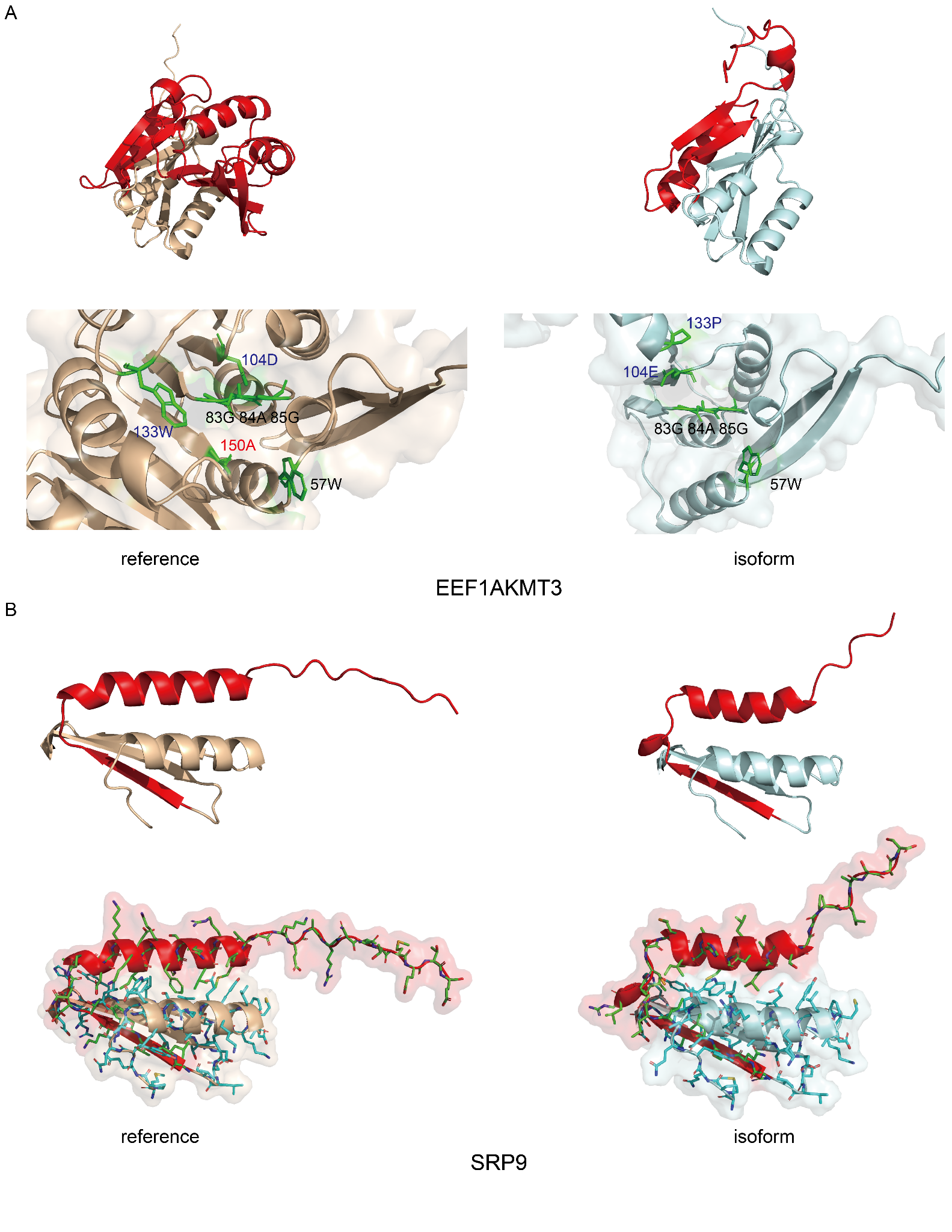
**Fig. S5: Example isoforms with low sequence identity.** (**A**) Top: reference and isoform structures for EEF1AMT3. Bottom: S-adenosyl-L-methionine binding sites in the EEF1AMT3 reference and isoform. Binding sites are shown as green sticks, with conserved sites labeled in black, replaced sites in blue, and lost sites in red. (**B**) Top: reference and isoform structures for SRP9. Alternative splicing regions are colored in red. Bottom: residue stick view to show the position of residues in reference and isoform structures. Residues in alternative splicing regions are shown in green sticks and residues in non alternative splicing regions are shown in blue sticks.


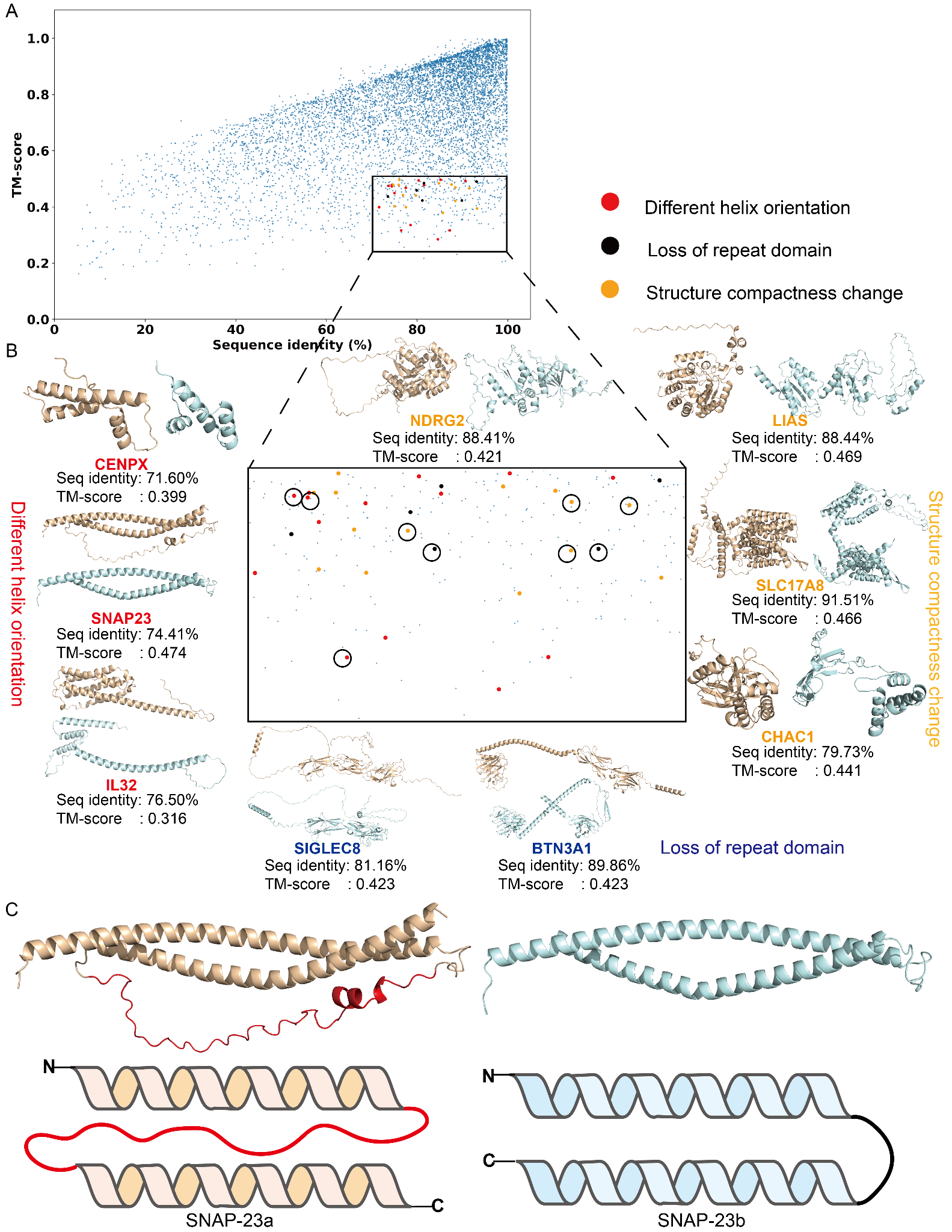


**Fig. S6: Overview of high sequence identity but low TM-score isoforms.** (**A**) Scatter plot of percent sequence identity between reference and alternate isoform (x-axis) vs. TM- score (y-axis). (PCC=0.565). Proteins with high TM-score but low sequence identity are shown in the box and colored by type of structural change. (**B**) Examples of isoforms with high sequence identity (>70%) but low TM-score (<0.5) categorized into three classes: structure compactness change, different helix orientation and loss of repeat domain. The middle box plot shows a zoomed-in view of the box in **Fig. S6A**. (**C**) Structures and simplified diagrams for SNAP23. The reference (SNAP-23a) is shown in wheat color, and the isoform (SNAP-23b) is shown in pale cyan. Alternatively spliced regions are colored in red.


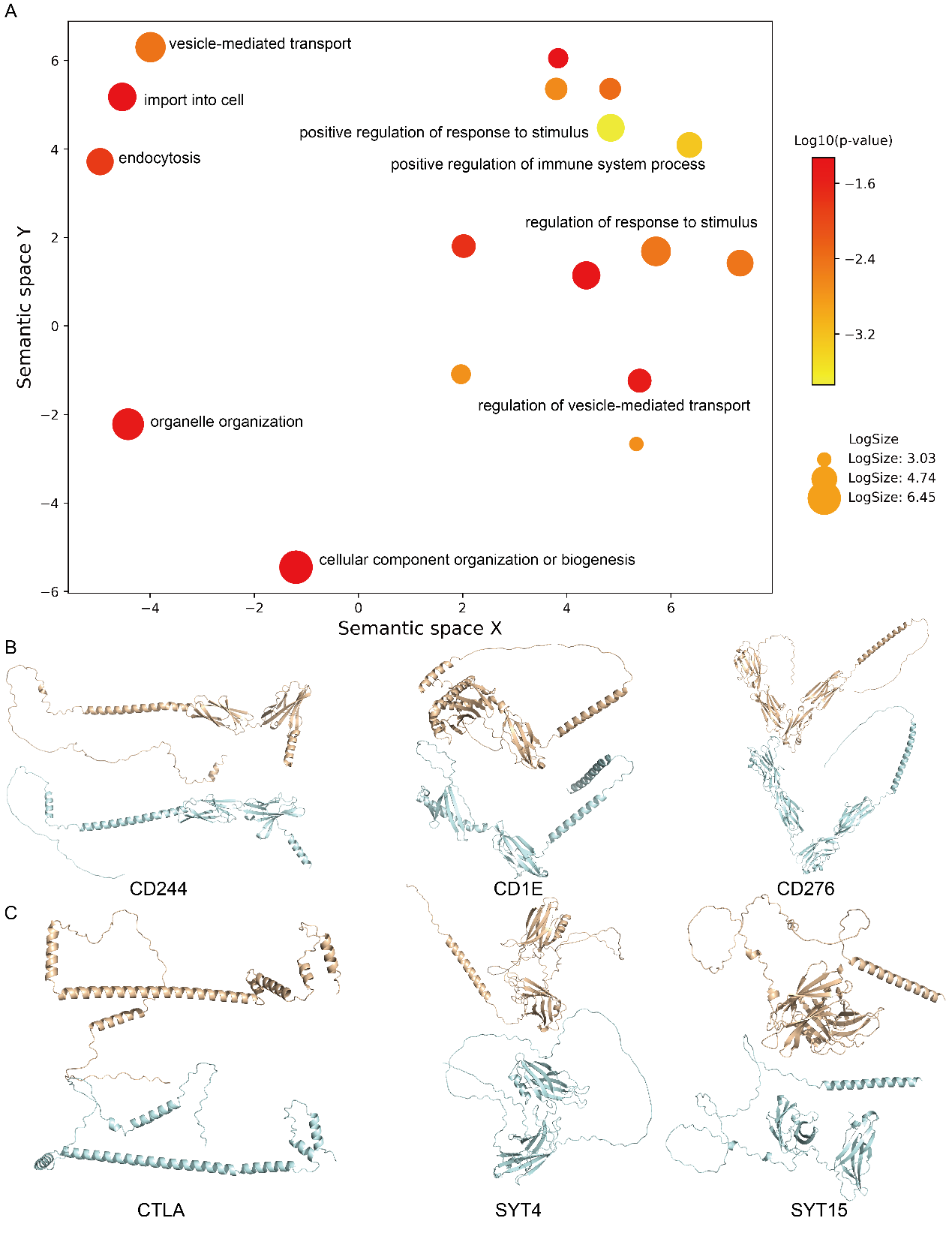


**Fig. S7: Loop-affected high sequence identity but low TM-score examples.** (**A**) Gene ontology analysis for loop-affected splicing isoforms, main GO terms are labeled, colored by the log10 p-value from the gene enrichment test. (**B**) Structures for immune response related examples: CD244, CD1E and CD276. (**C**) Structures for protein transport related examples: CTLA, SYT4 and SYT15.


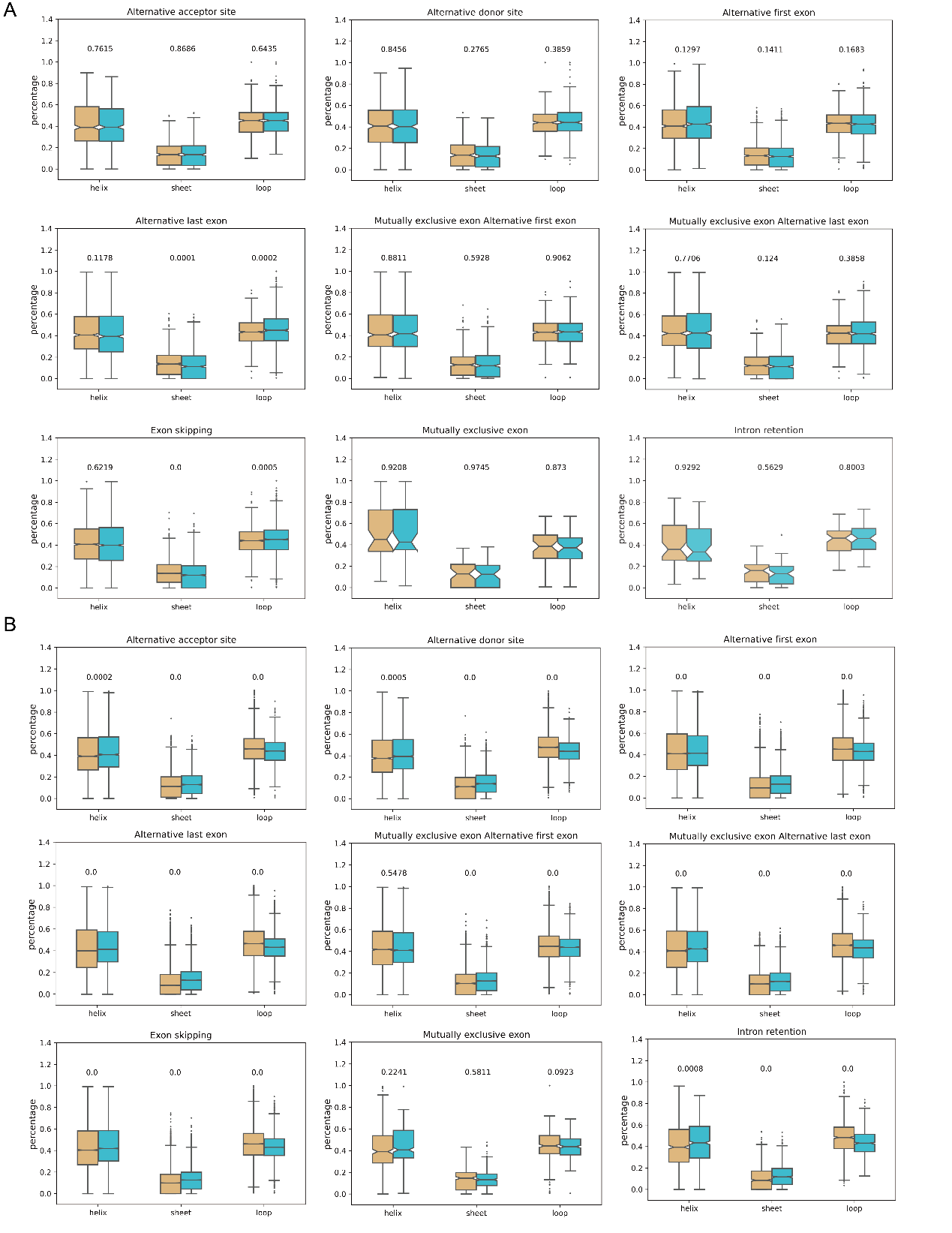


**Fig. S8: Secondary structure percentage for SwissProt dataset (A) and CHESS dataset (B).** P-value labeled for each secondary structure type percentage (helix, sheet and loop) between isoform and reference based on Mann–Whitney U test.

**
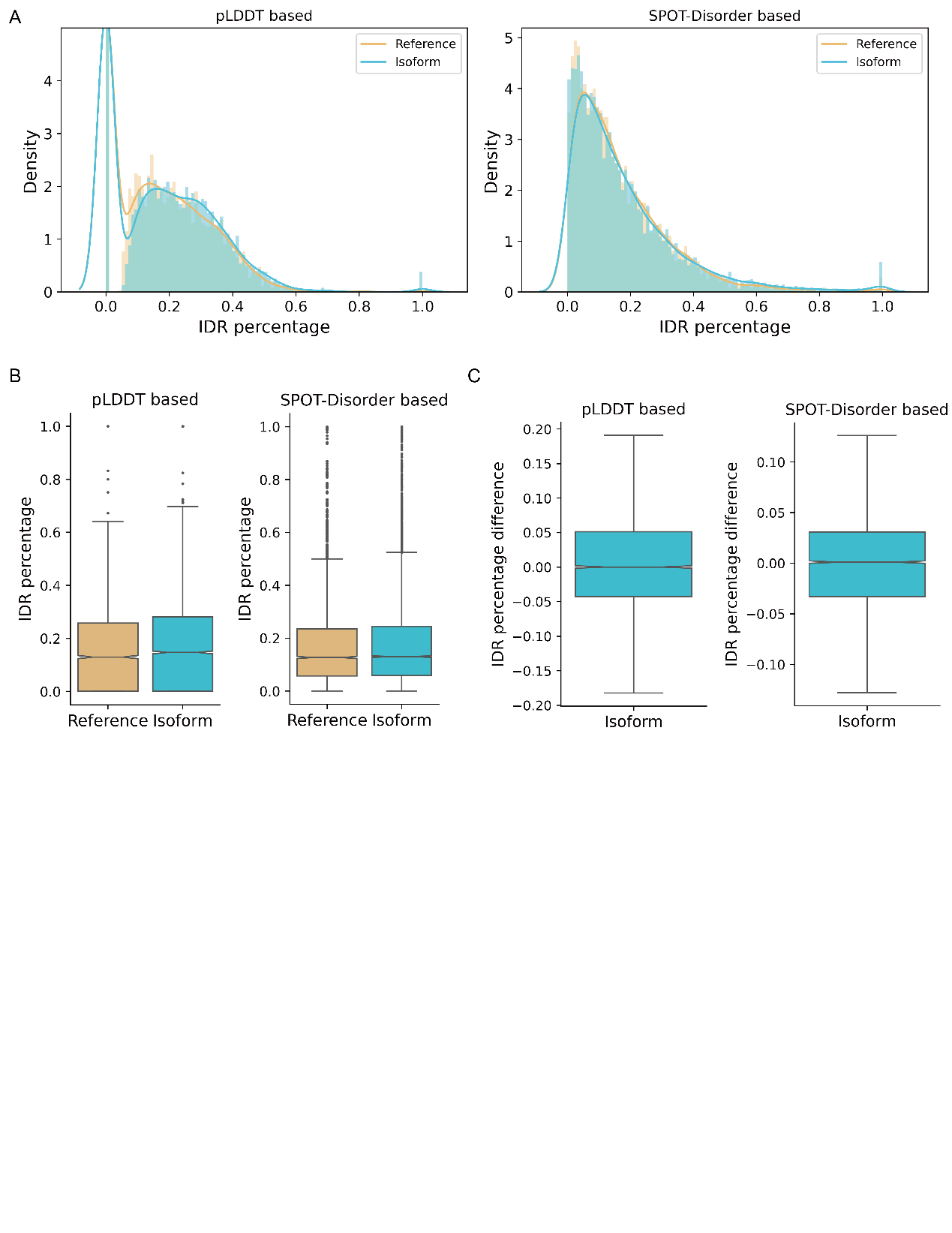
**

**Fig. S9: IDR percentage analysis in reference and isoform proteins.** (**A**) Density plot of IDR percentage for 4,450 reference and 7,630 isoform proteins calculated using window-averaged pLDDT scores and SPOT-Disorder. (**B**) Box plot of IDR percentage for references and alternative isoforms based pLDDT scores (p-value: 6.875e-5, Mann–Whitney U test) and based on SPOT-Disorder (p-value: 0.169, Mann–Whitney U test). (**C**) Differences in IDR percentage based on window-averaged pLDDT scores and SPOT-Disorder.


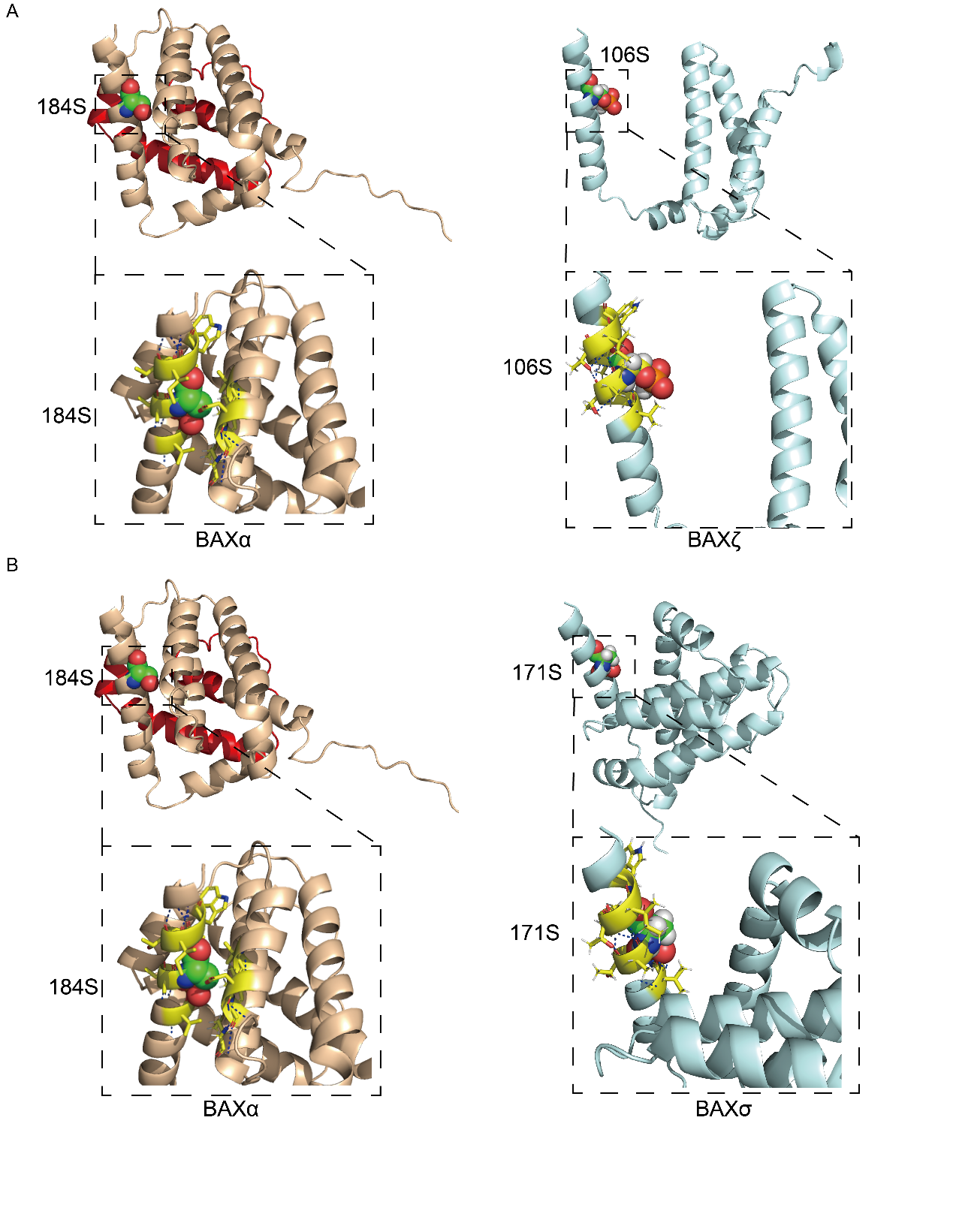


**Fig. S10: Surrounding environment change of post translational modification (PTM) sites of BAXζ and BAXσ.**  (**A**) Residue 184S is buried in BAXα (RSA: 1.18Å^2^), and the corresponding PTM site 106S is exposed in BAXζ (RSA: 51.55Å^2^). The residues within 4Å of the PTM site are represented in yellow sticks, and the polar contacts with those residues are represented in blue lines. (**B**) Residue 184S is buried in BAXα (RSA: 1.18Å^2^), and the corresponding PTM site 171S is exposed in BAXσ (RSA: 53.52Å^2^).


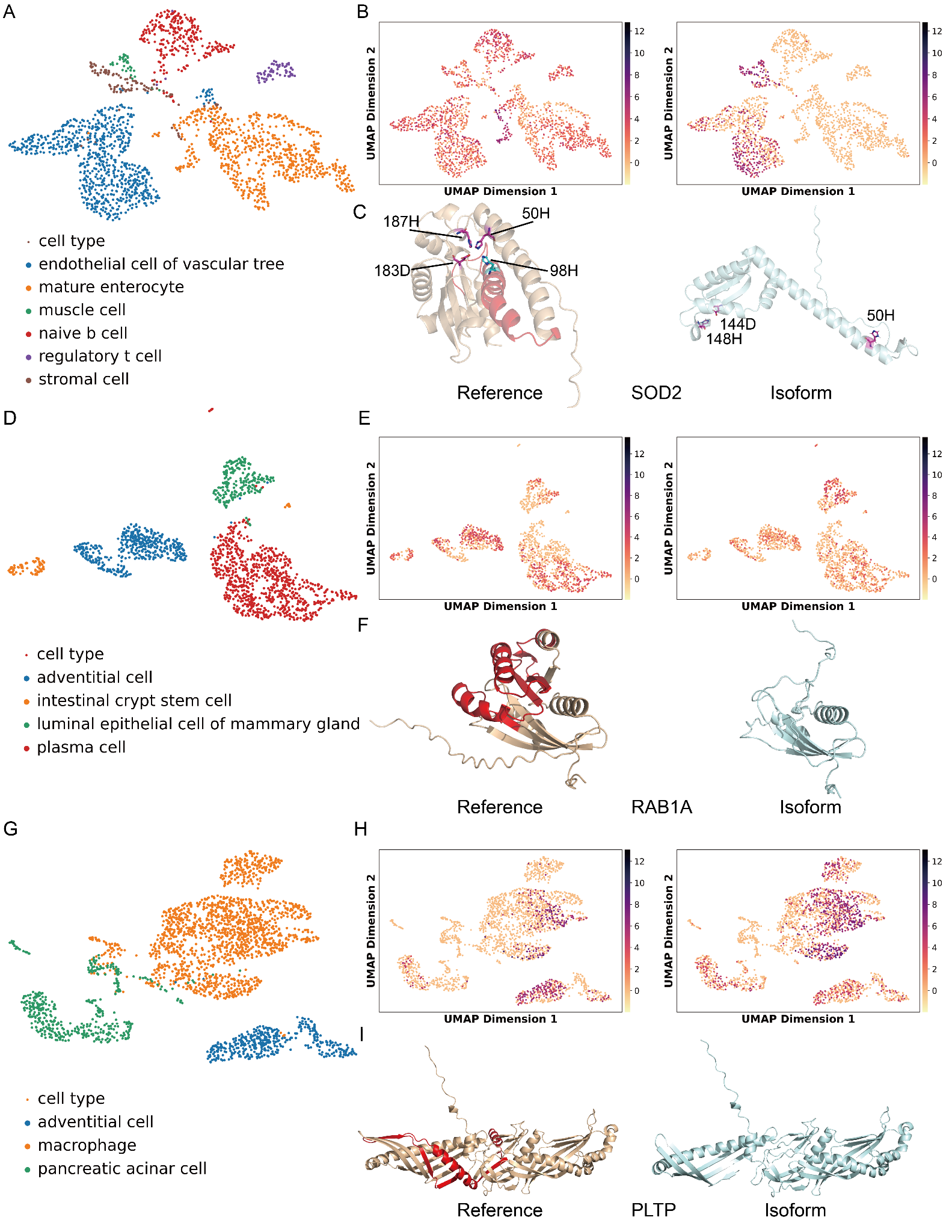


**Fig. S11: Isoform usage shift examples discovered from scRNA-seq data.** Cell type clusters with isoform usage shift for SOD2 (**A**), RAB1A (**D**) and PLTP (**G**) represented by UMAP plot. Expression of reference (left) and isoform (right) for SOD2 (**B**), RAB1A **(E**) and PLTP (**H**). Reference and isoform structures for SOD2 (**C**), RAB1A (**F**) and PLTP (**I**). In **Figure S11C**, we label the Mn^2+^ binding sites in SOD2 structures, and the binding sites retained in isoform (50H, 183D and 187H) are presented in pink sticks, and the binding site 98H lost in the SOD2 isoform is presented in cyan stick in reference.


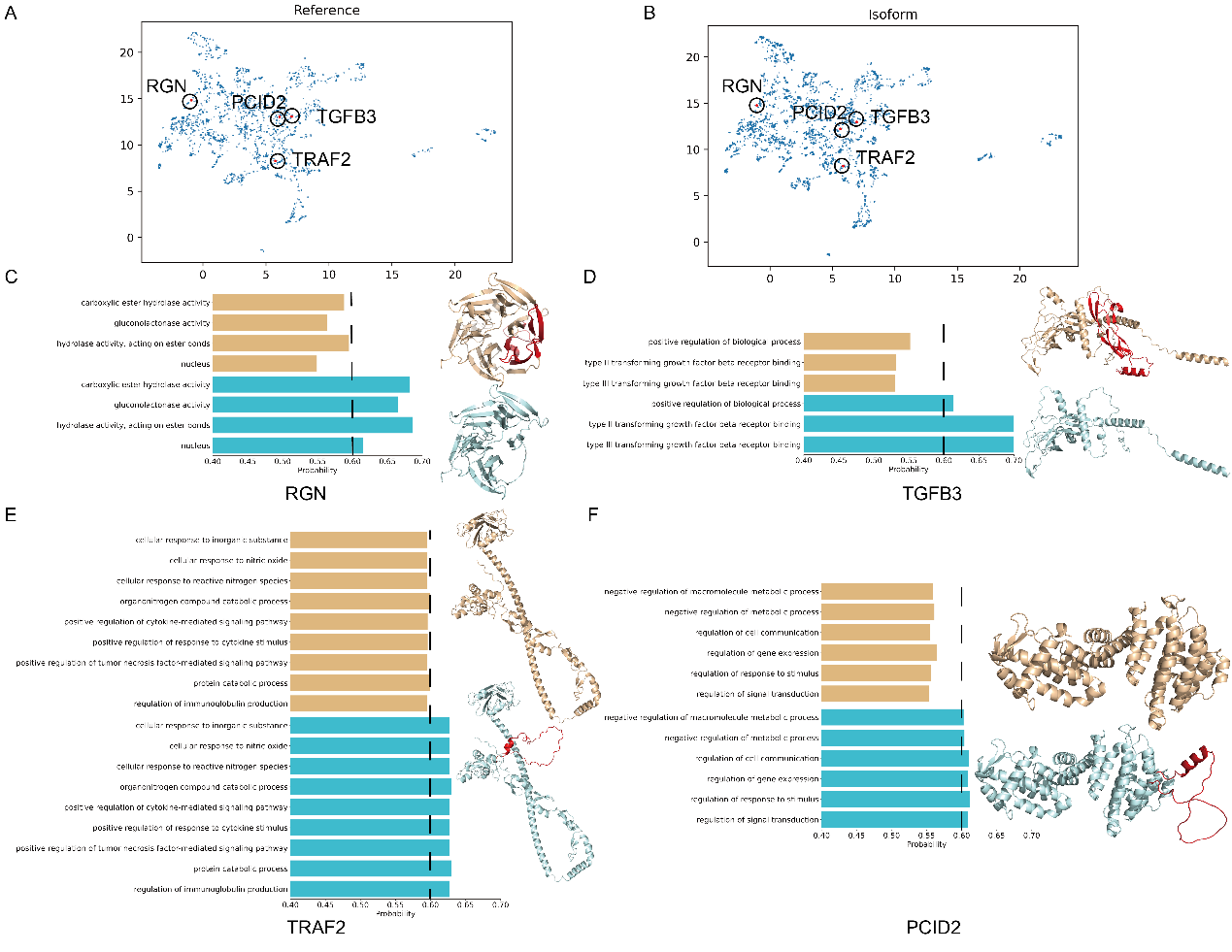


**Fig. S12: Examples of gain of function spliced isoforms.** UMAP plot based on jaccard distance for reference (**A**) and isoform (**B**), and the gain of function examples are circled. Structures and the predicted gain-in go terms for RGN (**C**), TGFB3 (**D**), TRAF2 (**E**) and PCID2 (**F**), the alternative splicing regions are colored in red in the structures.
